# Unveiling the enigmatic roles of basophils in HIV infection: A narrative review

**DOI:** 10.1097/MD.0000000000040384

**Published:** 2024-11-01

**Authors:** Emmanuel Ifeanyi Obeagu, Getrude Uzoma Obeagu, Callistus Adewale Akinleye

**Affiliations:** a Department of Medical Laboratory Science, Kampala International University, Kampala, Uganda; b School of Nursing Science, Kampala International University, Kampala, Uganda; c Department of Community Medicine, Osun State University, Osogbo, Nigeria.

**Keywords:** AIDS, basophils, cytokines, HIV, immunity

## Abstract

The intricate interplay between the human immunodeficiency virus (HIV) and the immune system has long been a focal point in understanding disease progression. Among the myriad of immune cells, basophils, often overshadowed, have recently emerged as pivotal contributors to the complex immunological landscape of HIV infection. This paper aims to provide a succinct overview of the enigmatic roles of basophils in HIV pathogenesis, elucidating their multifaceted functions and implications. Basophils, conventionally perceived as minor players in immune responses, exhibit active participation in HIV infection. Their activation triggered by viral antigens, cytokines, and immune complexes orchestrates a cascade of immune events, influencing immune modulation, cytokine release, and the activation of adaptive immune cells. Furthermore, basophils function as antigen-presenting cells, potentially impacting viral dissemination and immune dysregulation. Additionally, basophils serve as crucial regulators in HIV infection through cytokine secretion, notably interleukin (IL)-4, IL-13, and IL-3, influencing immune cell differentiation, polarization, and antibody production. Their interactions with various immune cells intricately shape the immune response against HIV, impacting disease progression and immune equilibrium. Moreover, harnessing basophils as potential vaccine targets or immune modulators represents a compelling avenue for future research. In conclusion, the emerging understanding of basophils’ multifaceted involvement in HIV infection challenges prior perceptions and underscores their significance in shaping immune responses and disease outcomes. This abstraction highlights the need for continued research to unlock the full potential of basophils, paving the way for novel strategies in combatting HIV/AIDS.

## 1. Introduction

The human immunodeficiency virus (HIV) remains a formidable global health challenge, intricately entwined with the intricate dynamics of the human immune system.^[1]^ Over the years, exhaustive research has elucidated numerous facets of immune responses orchestrated in response to HIV infection.^[2]^ However, amid the myriad of immune cells involved, basophils, traditionally relegated to the sidelines, have emerged as enigmatic participants in shaping the immunological landscape of HIV infection.^[3]^ Historically considered peripheral players in the realm of immune defense, basophils have recently garnered attention for their potential roles in modulating immune responses during HIV infection.^[4]^ Their overlooked status belies the intricate functionalities these cells possess, which have gradually surfaced through recent scientific inquiry. Contrary to earlier assumptions of passivity, basophils are now recognized as active participants in immune cascades triggered by HIV antigens, cytokines, and immune complexes.^[5]^

The significance of basophils in the context of HIV infection extends beyond their activation pathways.^[6]^ These cells, equipped with granular content and phenotypic markers, have unveiled their capacity to act as antigen-presenting cells, potentially influencing viral dissemination and immune regulation. Moreover, their ability to modulate immune responses through the secretion of cytokines such as interleukin (IL)-4, IL-13, and IL-3 underscores their regulatory role in shaping immune cell differentiation, polarization, and antibody production. Understanding the multifaceted roles of basophils in HIV infection holds immense promise for shedding light on hitherto unexplored avenues of disease progression and immune modulation. This exploration not only challenges traditional paradigms but also presents novel opportunities for therapeutic interventions. Targeting basophil-specific pathways may offer innovative strategies to modulate immune responses and mitigate HIV pathogenesis, potentially reshaping the landscape of HIV/acquired immunodeficiency syndrome (AIDS) management. In light of these evolving perspectives, this review aims to comprehensively delineate the intricate roles of basophils in HIV infection. By synthesizing current knowledge and emerging research, this exploration seeks to underscore the significance of basophils in shaping immune responses, thereby paving the way for novel therapeutic strategies in combating HIV/AIDS.

## 2. Basophils

Basophils are a type of white blood cell, belonging to the granulocyte family, and constitute a minor proportion of circulating leukocytes in the bloodstream. They play crucial roles in immune responses, primarily associated with allergic reactions and defense against parasites. Basophils are characterized by their distinctive large granules containing various compounds, notably histamine and other mediators such as leukotrienes and cytokines.^[7]^ These cells are typically found in the bloodstream but can also migrate to tissues in response to inflammatory signals. When activated, basophils release their granular contents into the surrounding environment. Histamine, in particular, is a potent mediator that contributes to allergic responses by inducing vasodilation, increasing vascular permeability, and attracting other immune cells to the site of inflammation.^[8]^

Basophils express surface receptors, including high-affinity IgE receptors, which bind to immunoglobulin E antibodies. This interaction primes basophils for activation upon reexposure to specific allergens, triggering the release of their granular contents and initiating allergic reactions.^[9]^ Beyond their role in allergic responses, recent research has unveiled previously unrecognized functions of basophils in various immune processes. They have been implicated in modulating adaptive immune responses by interacting with other immune cells, presenting antigens, and secreting cytokines that influence the behavior of T cells and other leukocytes. Though less abundant than other immune cells, the multifaceted roles of basophils in both normal immune function and disease contexts continue to be an active area of research.

## 3. Human immunodeficiency virus

Human immunodeficiency virus is a retrovirus that attacks the immune system, specifically targeting CD4+T cells, a type of white blood cell crucial for the body’s defense against infections. HIV progressively weakens the immune system, leading to acquired immunodeficiency syndrome AIDS if left untreated.^[10–12]^ The virus primarily spreads through contact with certain bodily fluids, including blood, semen, vaginal fluids, and breast milk. Common transmission routes include unprotected sexual intercourse, sharing contaminated needles or syringes, and transmission from an infected mother to her child during pregnancy, childbirth, or breastfeeding.^[13–15]^ Upon entering the body, HIV targets CD4+ T cells, where it replicates and causes gradual depletion of these immune cells. As the number of CD4+ T cells decline, the immune system becomes compromised, making the individual increasingly susceptible to opportunistic infections and certain cancers. The progression from initial HIV infection to AIDS can take years, during which time individuals may experience few or no symptoms.^[16–18]^

HIV infection is typically categorized into stages:

1. Acute HIV infection: This stage occurs shortly after initial exposure and is often associated with flu-like symptoms, including fever, fatigue, sore throat, and swollen lymph nodes. The virus multiplies rapidly during this phase.
2. Clinical latency: Also known as chronic or asymptomatic HIV infection, this stage is characterized by a lack of apparent symptoms. However, the virus continues to replicate at lower levels, steadily damaging the immune system.
3. AIDS: When the immune system becomes severely compromised, and the CD4+ T cell count falls below a certain threshold, the condition progresses to AIDS. At this stage, the individual becomes highly susceptible to opportunistic infections and may develop severe illnesses or cancers that are typically rare in people with healthy immune systems.

While HIV is a chronic condition, advancements in antiretroviral therapy (ART) have transformed HIV management. ART consists of a combination of medications that suppress HIV replication, reduce the viral load in the body, and slow down disease progression. When taken consistently and correctly, ART can effectively control the virus, allowing individuals with HIV to live longer, healthier lives and reducing the risk of transmitting the virus to others.^[19–21]^ Preventive measures, including the use of condoms, practicing safe injection practices, pre-exposure prophylaxis, and early diagnosis and treatment, play crucial roles in curbing the spread of HIV. Efforts toward raising awareness, reducing stigma, and promoting accessible healthcare are integral in addressing the global impact of HIV/AIDS.^[22–24]^

## 4. Basophil activation and HIV pathogenesis

Basophils, traditionally considered minor contributors to immune responses, have gained attention for their potential roles in HIV pathogenesis. Although the exact mechanisms are still being elucidated, research suggests that basophils can be activated during HIV infection and might play diverse roles in shaping the immune response to the virus.^[25]^ Activation of basophils in HIV infection occurs through various mechanisms. Viral antigens, immune complexes, and cytokines released during HIV infection can stimulate basophil activation. Upon activation, basophils release an array of mediators, including histamine, leukotrienes, cytokines (such as IL-4, IL-13), and chemokines, which can influence nearby immune cells and modulate the immune microenvironment.^[26]^ One significant aspect of basophil activation in HIV pathogenesis is their potential role as antigen-presenting cells. Basophils have been observed to capture and present HIV antigens to CD4+ T cells, which could potentially contribute to viral dissemination and immune dysregulation.^[27]^ This interaction between basophils and T cells may impact the immune response and affect disease progression. Moreover, basophils can secrete cytokines and chemokines that influence the activation and function of other immune cells. For instance, their release of IL-4 and IL-13 may affect the polarization of T cells, B cells, and other leukocytes, potentially shaping the adaptive immune response against HIV.^[28]^ Understanding the complex interactions between basophils and the immune response to HIV could offer insights into novel therapeutic strategies. Targeting basophil-specific pathways or manipulating their interactions with other immune cells may present opportunities for modulating immune responses and potentially altering disease outcomes. Further research into the specific mechanisms and functional consequences of basophil activation in HIV infection is necessary to fully comprehend their contributions to the pathogenesis of the virus.

## 5. Basophil-mediated immune regulation

Basophils, traditionally recognized for their roles in allergic reactions and parasitic infections, have gained attention for their regulatory functions in immune responses, including their potential contributions to immune regulation in the context of HIV infection. These granulocytes modulate the immune microenvironment through the secretion of various mediators, including cytokines such as IL-4, IL-13, and IL-3. These cytokines play critical roles in immune cell differentiation, polarization, and antibody production, thereby influencing immune responses during HIV infection.^[29]^ IL-4 and IL-13, released by activated basophils, have been associated with promoting Th2-type immune responses and influencing B cell differentiation toward antibody production. Their impact on antibody class switching may have implications for the development of HIV-specific antibodies, potentially affecting the control or clearance of the virus.^[30]^ Basophils’ ability to secrete IL-3, which supports the survival and proliferation of various immune cells, might also influence the immune landscape during HIV infection. This cytokine could contribute to the maintenance and activation of immune cells involved in antiviral responses, affecting the overall immune regulation against HIV.^[31]^ Furthermore, basophils can interact with other immune cells, including dendritic cells and T cells, through direct cell–cell contact or by releasing soluble mediators. These interactions can shape the immune response by influencing antigen presentation, T cell activation, and the balance between pro-inflammatory and anti-inflammatory signals, potentially impacting the outcome of HIV infection.^[32]^ Understanding the regulatory functions of basophils in HIV infection may provide insights into novel therapeutic approaches. Targeting basophil-mediated pathways or manipulating their interactions with other immune cells could potentially be harnessed for modulating immune responses and developing new strategies for managing HIV/AIDS. Further research is crucial to unravel the specific mechanisms underlying basophil-mediated immune regulation in the context of HIV infection and to explore their therapeutic implications.

## 6. Therapeutic implications

Targeting basophil-specific pathways or functions presents an avenue for modulating immune responses during HIV infection. Manipulating basophil activation or cytokine secretion could potentially help regulate immune responses, potentially mitigating immune dysregulation and controlling viral replication.^[33]^ Harnessing basophils’ ability to present antigens to CD4+ T cells opens possibilities for developing innovative vaccine strategies. Understanding how basophils process and present HIV antigens could aid in designing vaccines that elicit more robust and targeted immune responses against the virus.^[34]^ Modulating basophil-derived cytokines, such as IL-4, IL-13, and IL-3, might offer therapeutic opportunities. Blocking or manipulating these cytokines could potentially influence immune cell differentiation, antibody production, and immune regulation, impacting disease progression.^[35]^ Integrating strategies that target basophils alongside current ART may potentially enhance treatment outcomes. Comprehending the interplay between basophils and the immune response could guide the development of combined therapies that better control viral replication and immune dysregulation.^[36]^ Exploring basophil-mediated immune modulation might offer insights into preventive strategies beyond vaccines. Understanding how basophils influence immune cell activation and responses could inform approaches to prevent initial HIV infection or limit disease progression.^[37]^ Exploring the potential of biological agents that specifically target basophils or their signaling pathways could open new avenues for therapeutic intervention. Such targeted therapies might offer more precise and tailored approaches to modulate immune responses against HIV. However, it is important to note that while the therapeutic implications of basophil involvement in HIV infection are promising, translating these findings into clinical applications requires further research. Understanding the precise mechanisms, potential side effects, and long-term effects of manipulating basophil functions in the context of HIV infection is crucial for developing safe and effective therapies. Continued investigation into the complexities of basophil functions, their interactions within the immune system, and their specific roles in HIV pathogenesis will be instrumental in realizing the full therapeutic potential of targeting basophils in the management and treatment of HIV/AIDS.

### 6.1. Interactions of basophils, monocytes, and macrophages in further propagating the transmission HIV

The HIV primarily targets cells of the immune system, including CD4+ T lymphocytes, which play a central role in coordinating immune responses. While basophils, monocytes, and macrophages are not the primary targets for HIV replication, their interactions with the virus and other immune cells can contribute to the propagation and transmission of the virus. Basophils are a type of white blood cell involved in allergic reactions and immune responses to parasites. They express CD4 receptors but have a limited role in HIV replication. Basophils can release inflammatory mediators, and their activation may contribute to immune activation and inflammation, which are associated with increased HIV replication and transmission. Monocytes express CD4 receptors and can be infected by HIV. They can serve as a reservoir for the virus and facilitate its transport to various tissues, including the central nervous system. Once in tissues, monocytes can differentiate into macrophages, which are more permissive to HIV infection. Infected macrophages can release virions and contribute to the spread of the virus within tissues. HIV infection of monocytes can lead to immune activation, releasing pro-inflammatory cytokines and chemokines, which may enhance viral replication and contribute to disease progression. Macrophages are a major target for HIV infection. They can harbor the virus for extended periods, serving as a long-term reservoir even in the presence of ART. Macrophages are found in various tissues, including the brain, lymph nodes, and gastrointestinal tract. Their infection contributes to the establishment of viral reservoirs in these anatomical compartments. Infected macrophages can release HIV particles, contributing to viral transmission. Additionally, they can transfer the virus to CD4+ T cells, further spreading the infection. Interactions between HIV, monocytes, and macrophages can lead to a positive feedback loop of immune activation and inflammation. Chronic immune activation is a hallmark of HIV infection and is associated with disease progression. Inflammatory signals can recruit more target cells, including CD4+ T cells, to sites of infection, creating an environment conducive to viral replication and transmission.^[30–32]^

### 6.2. How to handle interaction between basophils and monocytes in HIV

The interaction between basophils and monocytes in the context of HIV is a complex aspect of the immune response. While basophils are not primary targets for HIV infection, their activation and communication with monocytes can contribute to immune responses and inflammation, influencing the course of HIV infection. Explore potential pharmacological interventions to modulate basophil activation. Controlling basophil responses may help in reducing inflammatory signals that could contribute to immune activation and HIV replication. Investigate the use of anti-inflammatory agents to dampen basophil-mediated inflammatory responses, potentially mitigating the positive feedback loop that can occur in HIV infection. Develop therapeutic strategies to modulate monocyte activation and differentiation. This may involve targeting specific signaling pathways or immune checkpoints to regulate monocyte responses during HIV exposure. Effective suppression of viral replication with ART may help reduce the overall immune activation associated with HIV infection. Explore the potential use of immunomodulatory agents that can selectively target basophil and monocyte responses. These agents could help balance the immune system and prevent excessive inflammation. Enhancing protective immune responses while minimizing inflammatory reactions could be a valuable strategy. Since basophils produce IL-4 and IL-13, explore the potential use of anti-IL-4/IL-13 therapy to modulate basophil functions. This approach may impact the overall immune response and inflammation associated with HIV infection. Consider the potential for personalized medicine approaches. Understanding individual variations in immune responses, including basophil and monocyte interactions, may allow for tailored interventions based on the patient’s immune profile. Identify biomarkers associated with basophil and monocyte activation in HIV-infected individuals. Monitoring these biomarkers could provide insights into the progression of infection and the effectiveness of interventions.^[34,35]^

## 7. Future perspectives

Further dissecting the intricate mechanisms underlying basophil activation, antigen presentation, and cytokine secretion in the context of HIV infection is crucial. Advanced techniques and models could help uncover specific pathways and interactions that dictate basophil functions during viral exposure.^[38]^ Expanding research on targeted therapies aimed at modulating basophil functions holds promise. Novel immunotherapies designed to selectively manipulate basophil activity or interfere with their interactions within the immune milieu could emerge as innovative treatment approaches. Advancements in understanding the heterogeneity of basophil populations among individuals could pave the way for personalized therapies. Tailoring treatments based on an individual’s basophil response profile might enhance therapeutic efficacy and minimize adverse effects.^[39]^ Utilizing insights into basophil-mediated antigen presentation could drive the development of more effective HIV vaccines. Understanding how basophils process and present viral antigens might aid in designing vaccines that trigger targeted and long-lasting immune responses against HIV. Exploring synergistic effects between therapies targeting basophils and conventional antiretroviral treatments could enhance therapeutic outcomes. Investigating combined approaches might lead to more effective control of viral replication and immune dysregulation.^[40]^ Identifying specific basophil-related biomarkers associated with HIV disease progression or treatment response could facilitate diagnostic and prognostic tools. These biomarkers might serve as indicators of disease severity or guide treatment decisions. Translating laboratory findings into clinical applications is critical. Conducting clinical trials to evaluate the safety and efficacy of therapies targeting basophils in HIV-positive individuals will be essential to validate their therapeutic potential. Integrating research on basophils into global health initiatives aimed at preventing, diagnosing, and treating HIV/AIDS is imperative. Collaborative efforts between researchers, healthcare providers, and policymakers can facilitate the implementation of novel strategies into healthcare systems worldwide. Continued research, technological advancements, and interdisciplinary collaboration are fundamental for unraveling the full spectrum of basophil functions in HIV infection. Harnessing this knowledge could lead to groundbreaking advancements in HIV/AIDS management and the development of innovative therapeutic strategies.

## 8. Conclusion

The emerging understanding of basophils’ intricate involvement in HIV infection unveils a captivating and multifaceted aspect of immune responses against the virus. These enigmatic cells, long overlooked in the context of HIV pathogenesis, are now recognized for their diverse roles in modulating immune regulation, antigen presentation, and cytokine secretion, thus influencing disease progression. The revelations regarding basophils’ activation pathways, antigen-presenting capabilities, and cytokine-mediated immune modulation offer promising insights into novel therapeutic interventions. Targeting specific basophil-related pathways or functions presents opportunities for manipulating immune responses, potentially reshaping the landscape of HIV/AIDS management.

Continued research endeavors focused on unraveling the complexities of basophil-mediated immune responses, coupled with collaborative efforts across scientific disciplines, hold the key to unlocking the full therapeutic potential of targeting basophils in combating HIV/AIDS. The integration of these insights into clinical practice and global health initiatives may herald a new era in the management of HIV infection, offering hope for improved treatments and preventive strategies on a global scale.

## Author contributions

**Conceptualization:** Emmanuel Ifeanyi Obeagu.

**Methodology:** Emmanuel Ifeanyi Obeagu, Getrude Uzoma Obeagu, Callistus Adewale Akinleye.

**Supervision:** Emmanuel Ifeanyi Obeagu.

**Validation:** Callistus Adewale Akinleye.

**Visualization:** Emmanuel Ifeanyi Obeagu, Callistus Adewale Akinleye.

**Writing – original draft:** Emmanuel Ifeanyi Obeagu, Getrude Uzoma Obeagu, Callistus Adewale Akinleye.

**Writing – review & editing:** Emmanuel Ifeanyi Obeagu, Getrude Uzoma Obeagu, Callistus Adewale Akinleye.
